# Spontaneous Perforated Uterine Pyometra Presenting With Abdominal Pain and Nausea

**DOI:** 10.7759/cureus.86129

**Published:** 2025-06-16

**Authors:** Xiu Zhang, Hao Bai, Tienan Feng

**Affiliations:** 1 Gynecology, Funan Hospital Affiliated to Fuyang Normal University, Fuyang, CHN; 2 Clinical Research Institute, Shanghai Jiao Tong University School of Medicine, Shanghai, CHN

**Keywords:** peritonitis, pneumoperitoneum, postmenopausal women, septic shock, spontaneous pyometra perforation

## Abstract

Pyometra perforation is a rare and severe gynecological acute abdomen. If diagnosis and treatment are not timely, it can lead to septic shock and even endanger the patient's life. Here, we present a 68-year-old postmenopausal woman who presented with symptoms of upper abdominal pain accompanied by nausea. CT indicated a large amount of free gas in the abdominal cavity and fluid in the abdominal and pelvic cavities. Laparotomy was performed considering gastrointestinal (GI) perforation. During the operation, it was diagnosed as perforation of pyometra. After the operation, the patient was transferred to the intensive care unit and died of septic shock 13 hours after the operation. For postmenopausal women with abdominal pain, pyometra perforation should be taken into consideration. Correct identification and timely treatment are particularly crucial for the patient's prognosis.

## Introduction

Pyometra is defined as an infectious disorder where purulent secretions accumulate within the uterus. The clinical incidence rate is approximately between 0.1% and 0.3%, but it is as high as 13.6% among postmenopausal women [1]. It is mostly due to low estrogen levels after menopause, atrophy, stenosis, and hardening of the cervical canal. It can also be seen in malignant diseases such as cervical cancer and endometrial cancer. The typical triad consists of purulent vaginal discharge, postmenopausal bleeding, and lower abdominal pain. However, some patients may lack purulent discharge or postmenopausal bleeding [2], just like this case. Gas on a CT scan is most commonly associated with a gastrointestinal (GI) perforation, but as this case demonstrates, it can sometimes be caused by a uterine perforation. This article details a case of a postmenopausal woman who developed septic shock due to pyometra perforation and eventually succumbed to the condition. The case offers valuable insights for the clinical diagnosis and treatment of such diseases.

## Case presentation

A 68-year-old postmenopausal woman presented with abdominal pain accompanied by nausea for more than two hours without any apparent cause. There was no fever or vomiting, vaginal bleeding, or fluid discharge, and her urination and defecation were normal. The patient has rheumatoid arthritis and has been on long-term non-steroidal anti-inflammatory drugs (NSAIDs). She denied other underlying medical histories, surgical history, and intrauterine manipulation history. Upon admission, her body temperature was 36.8°C, pulse rate was 95 beats per minute, respiratory rate was 21 breaths per minute, and blood pressure was 116/71 mmHg. Physical examination revealed mild abdominal distension with tenderness localized to the upper abdomen and periumbilical region, accompanied by muscular rigidity.

**Figure 1:**
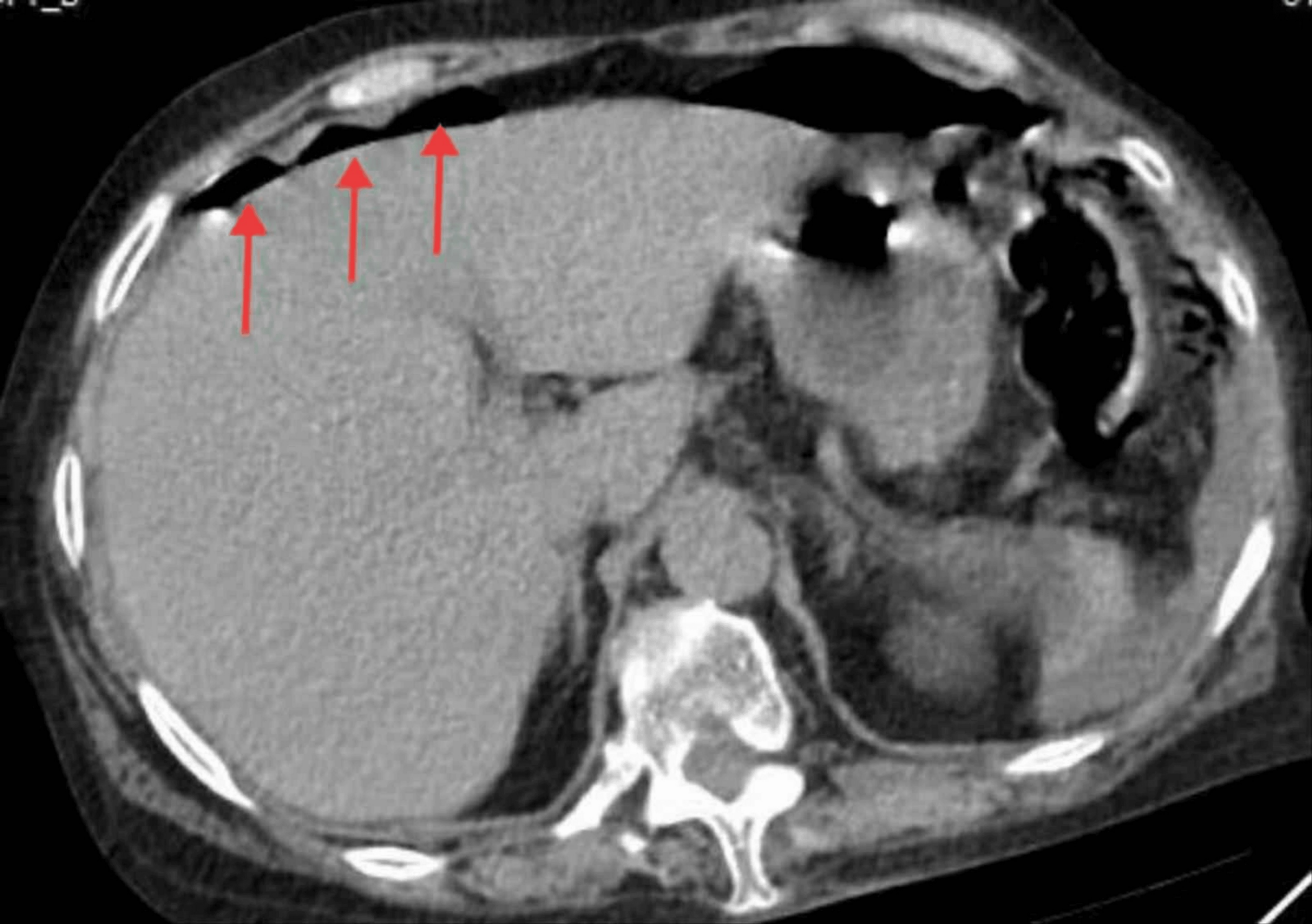
CT scan of the upper abdomen shows a large amount of free gas in the abdominal cavity

**Figure 2:**
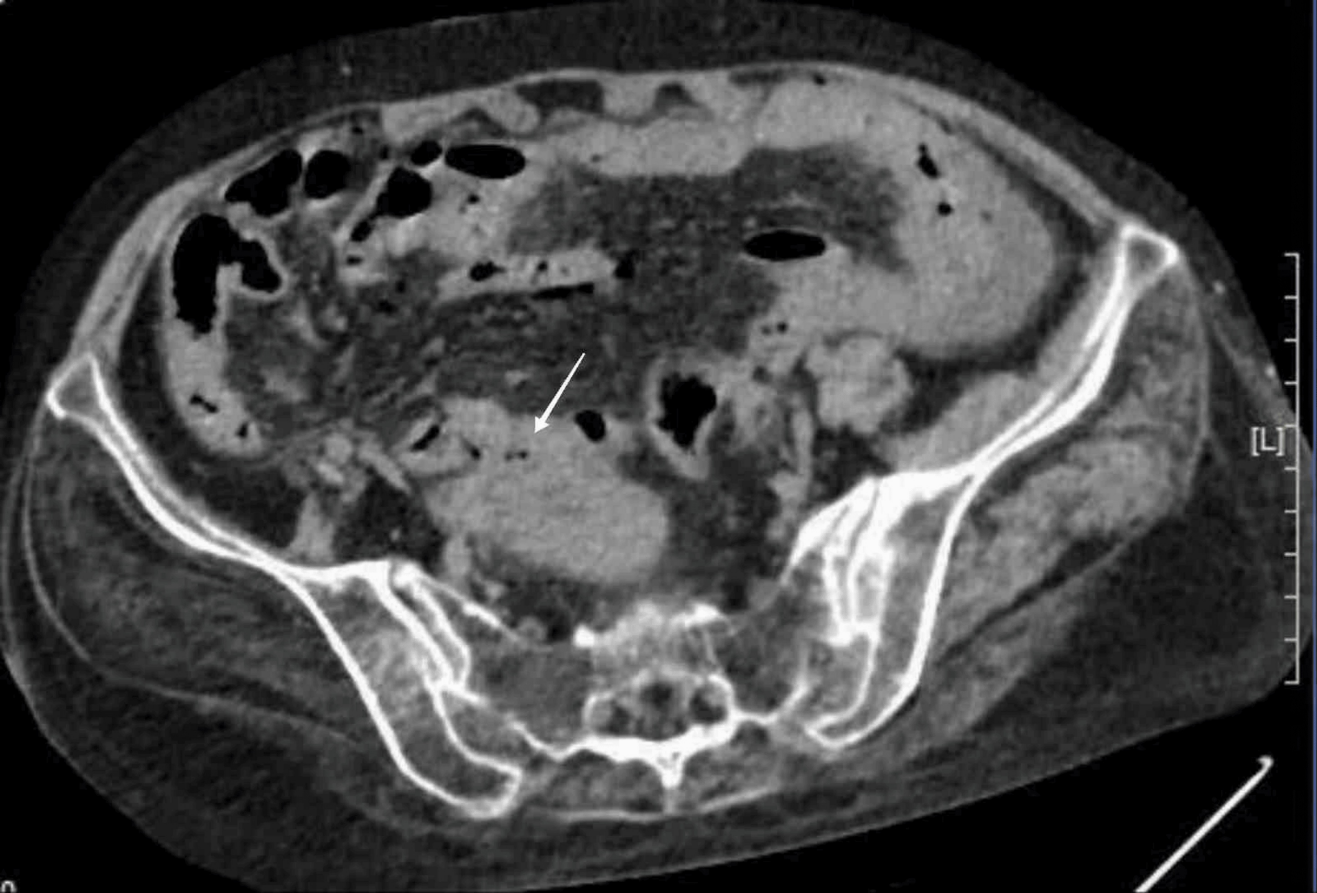
CT scan of the lower abdomen shows mixed-density shadow at the fundus of the uterus

Outpatient blood tests revealed a white blood cell count of 4.11×10^9^/L, a neutrophil count of 3.52×10^9^/L, and a hemoglobin level of 129 g/L. Electrolyte analysis showed a potassium level of 3.19 mmol/L and a glucose level of 22.23 mmol/L. The urinalysis results showed negative for urine ketone bodies. The electrocardiogram showed sinus rhythm, incomplete right bundle-branch block, and T-wave alterations. An upright abdominal plain film demonstrated localized intestinal pneumatosis. The CT scan shows a large amount of gas accumulation in the abdominal cavity (Figure 1), and there are shadows of mixed density around the uterus (Figure 2). The doctor considered GI perforation and recommended surgery, but the patient refused due to fear of surgical risks. As the abdominal pain continued to fail to relieve, the patient finally agreed to undergo exploratory laparotomy eight hours after admission. During the operation, 1,500 mL of purulent exudate was found in the abdominal cavity. The exudate was yellow in color and had a foul smell. A large amount of pus adhered to the edge of the liver, the splenic fossa, and the surface of the intestinal tract. A perforation with a diameter of approximately 1 cm was seen at the fundus of the uterus, which resembled a volcanic crater. Grayish-brown purulent fluid could be seen flowing out from the rupture site, emitting a foul smell. Both fallopian tubes and both ovaries were atrophied. Therefore, a transabdominal total hysterectomy plus bilateral adnexectomy was performed.

After surgery, the patient was transferred to the intensive care unit with an endotracheal tube in place. The blood pressure was 102/78 mmHg (maintained with norepinephrine), the heart rate was 119 beats per minute, and the patient was confused. Mechanical ventilation, fluid resuscitation, insulin for hypoglycemia, potassium supplementation, and imipenem 1 g intravenously every eight hours for anti-infection treatment were administered.

One hour after surgery, the blood pressure dropped to 82/60 mmHg, and the heart rate increased to 130 beats per minute. Repeated blood cell analysis showed: white blood cell count (WBC) of 13.52×10^9^/L, neutrophil count of 12.05×10^9^/L, and B-type natriuretic peptide (BNP) of 17,937 pg/mL. Liver function test results were albumin of 24.1 g/L and alanine aminotransferase (ALT) of 13 U/L. The electrolyte level was potassium (K⁺) of 4.12 mmol/L. Arterial blood gas analysis showed a pH of 7.19. A subclavian deep vein catheter was inserted, human albumin was infused, and amino acid-fat emulsion fluid resuscitation was initiated, with the remaining treatment plan unchanged.

**Figure 3:**
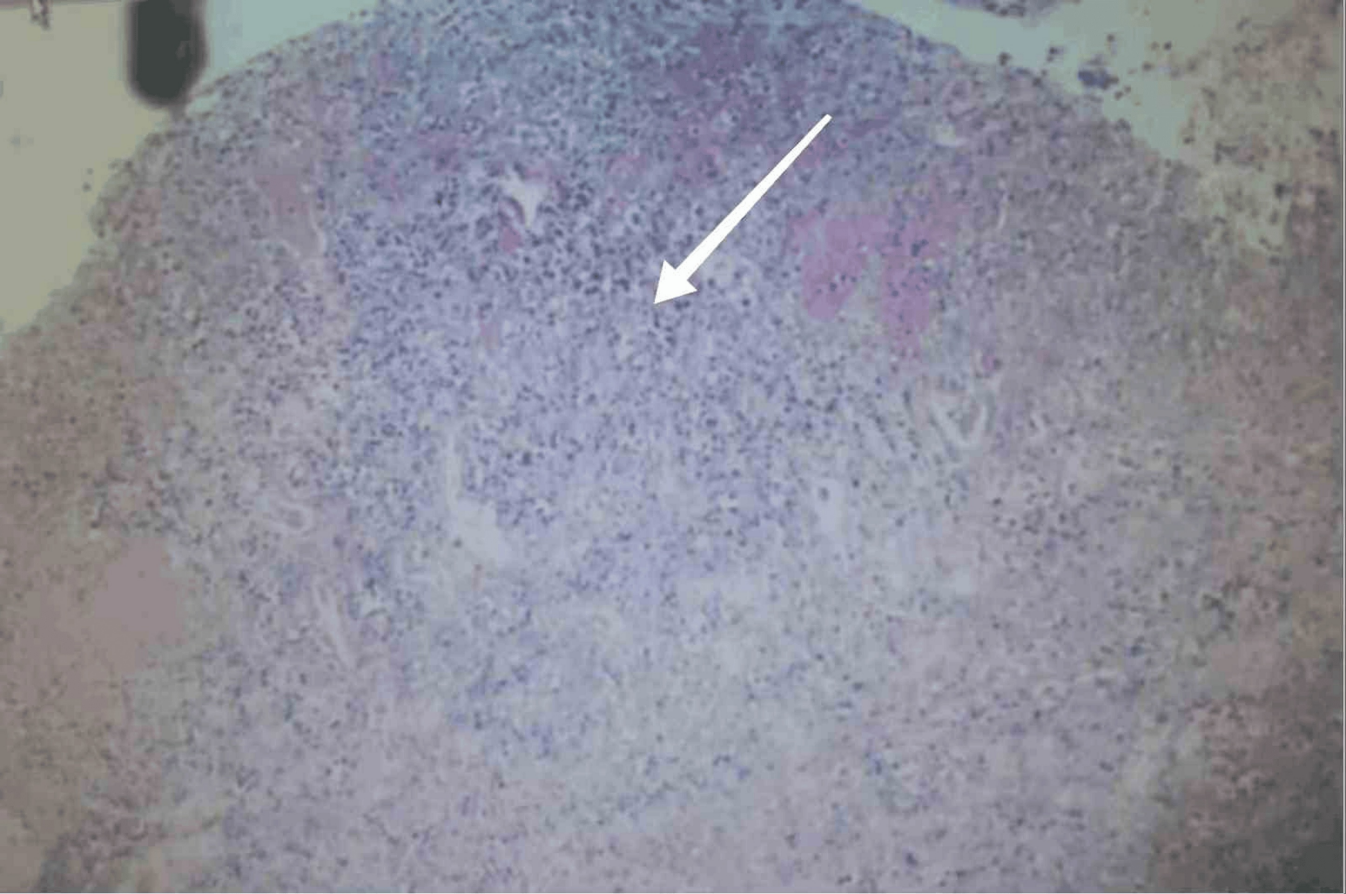
Histopathology of the endometrium shows the shedding of the mucosal epithelium, inflammatory exudation, and necrotic tissue

Eight hours after surgery, the patient was in a deep coma, with a blood pressure of 86/45 mmHg. A pump infusion of norepinephrine at 0.4 μg/(kg·min) was combined with metaraminol (2 μg/kg·min). The blood routine examination was rechecked again: white blood cell count was 17.29×10^9^/L, and BNP was 26,138 pg/mL. Digoxin was administered for cardiotonic effect, imipenem for anti-infection, and norepinephrine combined with metaraminol for blood pressure elevation. Thirteen hours after the operation, the patient suddenly experienced cardiac arrest and died due to circulatory failure caused by septic shock. Figure 3 shows that the postoperative pathological results suggest that the mucosal epithelium on the mucosal surface of the endometrium has shed, and there are inflammatory exudates, necrosis, and granulation tissues. A perforation is found at the fundus of the uterus, which, combined with the clinical situation, is consistent with the changes of uterine perforation. The results of pus culture taken during the operation: Escherichia coli, sensitive to imipenem.The results of blood culture: no bacterial growth after 24 hours of culture.

## Discussion

Reviewing the patient's symptoms, the patient presented with upper abdominal pain and discomfort around the umbilicus, accompanied by nausea upon admission, which is relatively rare in clinical practice. This may be related to a large accumulation of pus in the uterus, causing the uterus to enlarge beyond the pelvic cavity. No obvious abnormalities were found in the blood cell analysis and abdominal upright plain film. CT shows a large amount of fluid and gas in the abdominal cavity. This reminds clinicians that, for postmenopausal women presenting with abdominal pain, even if the results of the blood cell analysis are normal [3], they must be alert to the possibility of pyometra.

When the accumulation of pus leads to perforation, patients may experience severe abdominal pain, vomiting, fever, and other symptoms. It is easily misdiagnosed as GI perforation in clinical practice [4]. The author reviewed 15 cases of pyometra perforation since 2020 and found that the most common symptoms were abdominal pain (80%) [2,4-13], vomiting (33.3%) [5-7,12,13], fever (60%) [2,4,6-10,13,14], and symptoms of peritonitis. In some patients, free gas under the diaphragm could be seen in imaging examinations (46.6%) [5-8,10,13,14]; this reminds clinicians that this is not only a sign of GI perforation but may also be a perforation of a pyometra.

In some economically underdeveloped areas, clinicians also need to improve their communication skills to make patients aware of the severity of the disease and cooperate with the doctors' treatment to improve the prognosis of the disease. For postmenopausal women with a confirmed diagnosis of pyometra perforation, the preferred treatment option is total hysterectomy plus bilateral adnexectomy. However, some scholars have proposed that, for patients with septic shock, the principle of damage control should be followed to stabilize vital signs and create conditions for subsequent definitive repair surgery, and there have been successful treatment cases [10,15]. The clinical mortality rate of pyometra perforation is relatively high. Whether damage-control surgery should be adopted still needs further verification. Currently, there is insufficient data to compare the differences between the two treatment plans, which is also a direction for further research on the treatment measures of pyometra perforation in clinical practice.

During the clinical treatment process, the early diagnosis of pyometra perforation is another major challenge. For postmenopausal women with abdominal pain, transvaginal color-Doppler ultrasound examination should be completed, which helps identify pyometra. Decreased cognitive function could delay early diagnosis of pyometra and lead to septic shock and higher mortality [16]. When there is no perforation, performing cervical dilation and drainage, combined with antibiotics to control the infection, can lead to a better prognosis. Once uterine pyometra perforation is clinically diagnosed, laparotomy should be performed immediately. Pus specimens should be collected for culture to identify pathogenic microorganisms, and antibiotics should be adjusted based on the results of culture and drug sensitivity tests. The common pathogenic microorganisms of uterine pyometra are mainly Gram-negative bacilli and anaerobes, so the principle of early antimicrobial use should be based on broad-spectrum antibiotics. For patients with hemodynamic changes, multidisciplinary consultation should be conducted to formulate a treatment plan.

## Conclusions

For postmenopausal elderly women with abdominal pain, even if the blood cell analysis results are normal, clinicians should be highly vigilant for the possibility of pyometra and, when suspected, perform a transvaginal ultrasound examination. In addition, pyometra caused by cervical cancer or endometrial cancer should be excluded with appropriate testing. When a patient presents with symptoms of acute diffuse peritonitis and imaging shows free gas under the diaphragm, perforation of pyometra should be excluded. Early identification of pyometra is crucial for improving treatment prognosis.

Once pyometra with perforation is diagnosed, immediate surgery, peritoneal lavage, high-dose broad-spectrum antibiotics, and fluid resuscitation for shock should be initiated. The treatment of elderly patients requires collaboration from a multidisciplinary team. For patients with hemodynamic instability, the principles of damage control surgery can be adopted, using staged and simplified surgical procedures to control life-threatening injuries and gain time for subsequent treatment.
